# Super-Enhancer Associated Five-Gene Risk Score Model Predicts Overall Survival in Multiple Myeloma Patients

**DOI:** 10.3389/fcell.2020.596777

**Published:** 2020-12-03

**Authors:** Tingting Qi, Jian Qu, Chao Tu, Qiong Lu, Guohua Li, Jiaojiao Wang, Qiang Qu

**Affiliations:** ^1^Department of Pharmacy, Xiangya Hospital, Central South University, Changsha, China; ^2^Department of Pharmacy, The Second Xiangya Hospital, Central South University, Changsha, China; ^3^Institute of Clinical Pharmacy, Central South University, Changsha, China; ^4^Department of Orthopaedics, The Second Xiangya Hospital, Central South University, Changsha, China; ^5^Institute for Rational and Safe Medication Practices, National Clinical Research Center for Geriatric Disorders, Xiangya Hospital, Central South University, Changsha, China

**Keywords:** multiple myeloma, super-enhancer, LASSO, overall survival, risk score model

## Abstract

Multiple myeloma (MM) is a malignant plasma cell tumor with high heterogeneity, characterized by anemia, hypercalcemia, renal failure, and lytic bone lesions. Although various powerful prognostic factors and models have been exploited, the development of more accurate prognosis and treatment for MM patients is still facing many challenges. Given the essential roles of super-enhancer (SE) associated genes in the tumorigenesis of MM, we tried to initially screen and identify the significant prognostic factors from SE associated genes in MM by the least absolute shrinkage and selection operator (Lasso) penalized Cox regression, univariate and multivariate Cox regression analysis using GSE24080 and GSE9782 datasets. Risk score model of five genes including *CSGALNACT1*, *FAM53B*, *TAPBPL*, *REPIN1*, and *DDX11*, was further constructed and the Kaplan-Meier (K-M) curves showed that the low-risk group seems to have better clinical outcome of survival compared to the high-risk group. Time-dependent receiver operating characteristic (ROC) curves presented the favorable performance of the model. An interactive nomogram consisting of the five-gene risk group and eleven clinical traits was established and identified by calibration curves. Therefore, the risk score model of SE associated five genes developed here could be used to predict the prognosis of MM patients, which may assist the clinical treatment of MM patients in the future.

## Introduction

Multiple myeloma (MM) is an incurable plasma cell hematologic malignancy with high morbidity and mortality (Kumar et al., 2017; Bai et al., 2020). The median overall survival of MM patients is approximately 6 years (Durie et al., 2017) and the 5-year survival rate is currently 48.5% (Szudy-Szczyrek et al., 2020). With the continuous advances in treatment regimes, such as immunomodulatory drugs (IMiD) and proteasome inhibitors (PI), the life quality of MM patients has been improved (Witte et al., 2020; Xu et al., 2020). Bortezomib and dexamethasone combined with cyclophosphamide or adriamycin are considered to be the first-line therapy to improve efficacy (Gerecke et al., 2016). For relapsed MM patients, lenalidomide in combination with dexamethasone was effective to improve the overall survival (OS) of MM patients (Dimopoulos et al., 2009). More importantly, the addition of daratumumab in early relapses produced greater benefit in progression-free survival (PFS; Dimopoulos et al., 2016). However, since most MM patients eventually relapse and become refractory, patient heterogeneity, and complexity may continue to increase (Liu et al., 2020; Sanchez et al., 2020). In addition, the prognosis of MM patients is highly heterogeneous and individual differences, leading to survival ranging from several months to more than 10 years (Rajkumar, 2018; Çiftciler et al., 2020). Therefore, risk stratification and reliable prognostic biomarkers for MM patients are urgently needed.

Prognostic factors for MM patients include traditional clinical information [such as the stage of the disease, age, and comorbidities (Szudy-Szczyrek et al., 2020)], cytogenetic abnormalities, and other laboratory tests [such as neutrophils to lymphocytes ratio (NLR), platelets to lymphocytes ratio (PLR), serum lactate dehydrogenase (LDH), and electrocardiographic (ECG; Dimopoulos et al., 1991; Kumar et al., 2017; Szudy-Szczyrek et al., 2020; Wang J. et al., 2020)]. The International Staging System (ISS) and the revised International Staging System (R-ISS) were established to create a unified prognostic index, but incorporating other important prognostic factors into the current risk stratification systems is challenging (Ooi et al., 2016). Recently, prognostic models based on gene expression signature make it possible to predict risk stratification in newly diagnosed MM patients, and more importantly, it is even better in predicting OS than R-ISS (Liu et al., 2019; Bai and Chen, 2020).

The least absolute shrinkage and selection operator (Lasso) penalized Cox regression is a method for variable selection and shrinkage in Cox’s proportional hazards model proposed by Robert Tibshirani (Tibshirani, 1997). Currently, Lasso is widely used for the survival analysis of high-dimensional data (Jiang et al., 2018). Compared to the traditional stepwise regression, Lasso could reduce the number of variables, because some coefficients of less influential variables will become zero through regularization (Friedman et al., 2010). Since the most important and influential variables are retained, Lasso regression could still produce an accurate and refined model, even if it is less variable (Tibshirani, 1997; Gao et al., 2010). Wang et al. identified eleven important immune cell activation pathways most associated with the overall survival of MM patients among the 28 immune cell pathways by using Lasso regression analysis (Wang Y. et al., 2020). Using group Lasso, Li et al. (2020) screened eleven key risk factors of acute kidney injury in patients with hematologic malignancies, indicating the great significance of Lasso in clinical application.

Super-enhancers (SEs) are *cis*-regulatory elements, which are defined as a cluster of active enhancers that spans a large region of the genome (Jia et al., 2020). SEs are generally occupied with abundant signals of H3K4me1, H3K27ac, p300, Mediator, RNA polymerase II, BRD4, CDK7, and other master transcription factors, which are responsible for the tissue-specific gene expression (Wang et al., 2019). The recurrent loss or gain of SEs has been reported in various tumors (He et al., 2019) including MM, indicating the important role of SEs in MM (Qu et al., 2020). Lovén et al. (2013) identified 681 SE-associated genes, including some key MM genes such as *MYC*, *IRF4*, *PRDM1*, and *XBP1*. Jin et al. (2018) found 55 recurrent transcription factor (TF) associated SEs in MM samples and established a SE-TF regulatory network, which provided some critical TFs to target in MM. Besides, MM cell growth was driven by non-overlapping controlled transcription of promoters and SEs (Fulciniti et al., 2018). Therefore, screening and identifying more SE-associated genes as attractive therapeutic, diagnostic and prognostic targets are emergently needed in MM.

**FIGURE 1:**
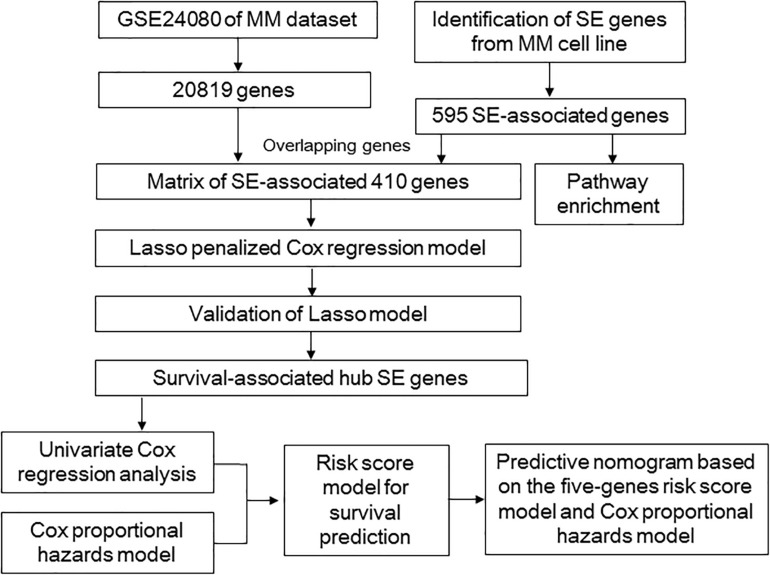
Overall design to develop and validate the prognostic model for multiple myeloma.

Given the critical roles of SE-associated genes in MM, in the present study, we tried to screen a cluster of SE-associated prognostic genes via Lasso, which was further used to construct a risk score model. This model was helpful for risk stratification and prognosis. Besides, the SE-associated gene risk group and several critical clinical indicators were included in a nomogram, which could provide a method to predict the OS of MM patients clinically. The overall design of our study was shown in Figure 1.

## Materials and Methods

### Data Source and Microarray Analysis

The microarray data and clinical data of GSE24080 (Shi et al., 2010) as the training dataset and GSE9782 (Mulligan et al., 2007) as the validation dataset were downloaded from Gene Expression Omnibus (GEO) database^1^. GSE24080 containing 559 untreated MM samples and GSE9782 containing 528 samples were conducted by GPL570 and GPL96/GPL96, respectively.

### Pathway Enrichment of SE-Associated Genes

Super-enhancers and their related genes identified from the MM1S cell line were downloaded from the website http://dbcorc.cam-su.org. H3K27ac ChIP-seq signal was also used to screen SE-associated genes in the MM1S cell line. Pathway enrichment analysis was performed to uncover the biological function of SE-associated genes^2^. Genes in GSE24080 dataset and the SE-associated genes in the MM1S cell line were overlapped to obtain the matrix of SE-associated genes.

### Lasso Penalized Cox Regression Analysis

Super-enhancer-associated genes were further analyzed through the Lasso penalized Cox regression to narrow and select the potential prognostic genes. The contributions of all the genes were weighted by their relative coefficients. Ten-fold cross-validation was used to derive the best-fit lambda value to minimize the mean cross-validated error through the R package “glmnet.” Two parameters of lambda.min and lambda.1se were chosen to establish two ideal prognosis models. The Wilcoxon tests were used to distinguish death and survival events based on the two ideal models. Receiver operating characteristic (ROC) curves were plotted and the area under the ROC (AUC) was also calculated to compare the performance of the two Lasso models.

### Establishment of a Risk Score Model to Predict Patient Overall Survival

After Lasso penalized Cox regression analysis was performed, the risk score model of selected genes was established and the risk scores of all samples were calculated according to the equation: risk score = Σβi ^∗^ Xi. Xi is the gene expression level and βi is the regression coefficient value. Patients were divided into high-risk (risk score > 0) group and low-risk (risk score < 0) group. Gene expression profiles of the identified SE-associated genes were plotted by the heatmap according to the risk group.

### Survival Analysis and Time-Dependent ROC

Kaplan-Meier (K-M) curves were plotted and log-rank tests were performed to compare the overall survival between the high-risk and low-risk groups of MM patients by R packages “survival” and “survimer.” Log-rank *p* value less than 0.05 was considered statistically significant. Time-dependent ROC curves were plotted and AUC values were calculated to evaluate the discriminatory ability of the risk score model.

### Univariate and Multivariate Cox Regression Analyses

Univariate and multivariate Cox regression were conducted to analyze the SE-associated genes and clinical features including treatment protocol (PROT), AGE, SEX, RACE, ISOTYPE, Beta-2 microglobulin (B2M), C-reactive protein (CRP), Creatinine (CREAT), Lactate dehydrogenase (LDH), Albumin (ALB), Haemoglobin (HGB), Aspirate plasma cells (ASPC), Bone marrow biopsy plasma cells (BMPC), magnetic resonance imaging (MRI), Clinical parameter S1 (CPS1), Clinical parameter R1 (CPR1), and cytogenetic abnormalities (Cyto_Abn) by R packages “survival” and “survimer.” The *p* value, hazard ratio and 95% confidence intervals for each variable were also calculated.

### Building and Validating the Predictive Nomogram

A nomogram containing twelve prognostic predictors (eleven clinical indicators and the five-gene risk group) was conducted to predict the 1-year, 3-year, 5-year, and 7-year overall survival of MM patients by R package “rms.” Meanwhile, to compare the independently predictive effect of the five-gene risk group, we also built another nomogram containing only eleven clinically indicators but no five-gene risk group. Total points were calculated by adding the point of every indicator. The predicted overall survival probability can be obtained by drawing a straight line from the Total Points. Concordance-index (C-index) was also calculated to evaluate the performance of nomogram by R package “Hmisc.” Calibration plots were generated by a plot of the actual survival probabilities against the predicted survival probabilities, which were used to visualize the performances of the nomograms. The 45° gray line represented the ideal prediction performance of the nomogram. One sample from GSE24080 was randomly selected to verify the probability of 1 to 7-year overall survival based on these prognostic predictors in the nomogram. The total points of variables were calculated by the R package “nomogramEx.” The interactive nomogram was calculated by the function of R package “glm” and showed using “regplot.”

## Results

### Identification of SE-Associated Genes in MM1S Cell Line

A total of 20819 genes in MM patients were obtained from the GSE24080 dataset. 662 SEs predicted in the MM1S cell line were downloaded from the website^3^ and then 595 SE-associated genes were identified. Pathway enrichment analysis indicated that these SE-associated genes were closely related to lymphocyte activation and the regulation of cytokine production (Supplementary Figures 1A,B). By overlapping the genes between the 20819 genes identified from the GSE24080 dataset and the 595 SE-associated genes, the matrix of SE-associated genes for MM was obtained. As a result, 410 SE-associated genes were screened and used in the following analysis.

### Construction of Lasso Penalized Cox Regression Model

**FIGURE 2:**
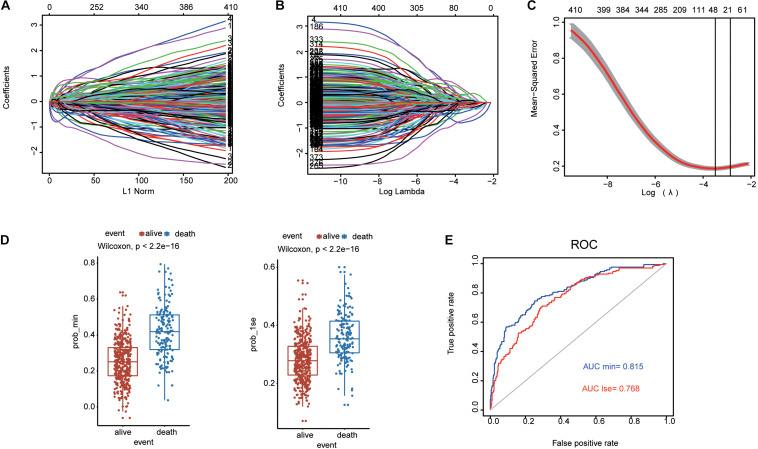
Lasso penalized Cox regression analysis of SE-associated 410 genes. **(A,B)** The coefficient values at varying levels of penalty. **(C)** The identification of best Lambda value. The left solid vertical line is the logarithm of lambda.min (35-gene group), the right solid vertical line is the logarithm of lambda.1se (13-gene group). **(D)** Two models based on lambda.lse and lambda.min are used to distinguish the survival and death events by the Wilcoxon test. **(E)** ROC curves are used to compare the predictive performance for prob-min and prob-1se to predict patient survival.

Lasso penalized Cox regression was conducted to further select potential prognosis-related genes among these 410 genes. The coefficient values for each gene at varying levels of penalty were calculated (Figures 2A,B). Genes with non-zero coefficients were considered to have strong prognostic potential in the Lasso penalized regression model. Ten-fold cross-validation was used to obtain the best lambda value. As a result, two lambda values (lambda.min and lambda.1se) were selected to rebuild two Lasso models, which produced two groups of genes (35-gene group of lambda.min and 13-gene group of lambda.1se; Figure 2C). The predictive performances of these two models were compared by the Wilcoxon test. As shown in Figure 2D, both the two predictive models could well distinguish the survival and death events (Wilcoxon test *p* = 2.2e-16). The AUCs of ROC curves of the two predictive models were 0.815 and 0.768, indicating that both of the two models were favorable for predicting overall survival (Figure 2E). Considering the predictive performances of two predictive models were not obviously different according to AUC and Wilcoxon test, we further investigated the 13-gene model.

### Development of 13-Gene Risk Score Model in MM Patients

**FIGURE 3:**
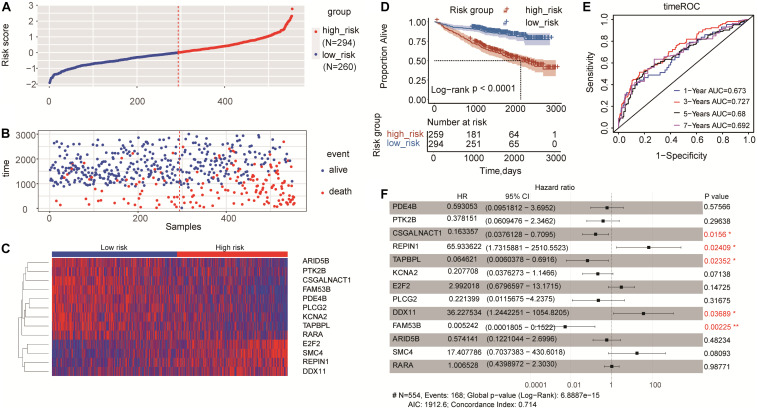
Risk score model based on 13-gene signature in MM. **(A)** 13-gene risk score distribution. Multiple myeloma patients were divided into the high-risk group and low-risk group based on the cut-off value. **(B)** The survival status and time in high-risk and low-risk groups. **(C)** The expression profiles of the 13 genes in high-risk and low-risk groups. **(D)** Kaplan-Meier analysis of the 13-gene risk score model to predict patient survival. Upper: Kaplan-Meier curve of the overall survival between the high-risk and low-risk groups. Lower: the number of patients at risk in the high-risk and low-risk groups at different time points. **(E)** Time-dependent ROC curves for the 13-gene model to predict patient survival. **(F)** Multivariate Cox regression analysis of the 13 genes (*p < 0.05 and **p < 0.01). Hazard ratio and 95% CI are showed in the figure. Global log-rank *p*, C-index and AIC were also calculated and showed.

According to the risk score of every patient predicted by the coefficient of Cox regression analysis, the patients were separated into the high-risk group (*n* = 294) and low-risk group (*n* = 260; Figure 3A). The events of death were more enriched in the high-risk group compared to that in the low-risk group (Figure 3B). A heatmap of gene expression levels indicated that nine genes were underexpressed and four genes were overexpressed in the high-risk group (Figure 3C). The K-M analysis showed that the high-risk group was an effective prognostic indicator for the inferior survival outcome (log-rank *p* < 0.0001; Figure 3D). Moreover, the time-dependent ROC analysis was conducted to evaluate the discriminatory ability of this 13-gene risk score model. The AUC values for 1-year, 3-year, 5-year, and 7-year survival were 0.673, 0.727, 0.68, and 0.692, respectively, (Figure 3E).

### Construction and Validation of the Five-Gene Risk Score Model

**FIGURE 4:**
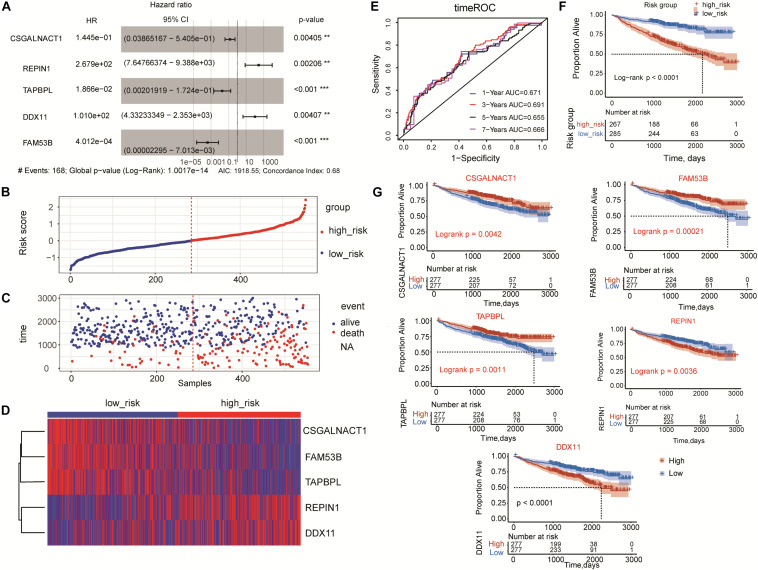
Risk score model based on five-gene signature in MM. **(A)** Multivariate Cox regression analysis of the five genes (**p < 0.01 and ***p < 0.001). **(B)** five-gene risk score distribution. Multiple myeloma patients were divided into the high-risk group and low-risk group based on the cut-off value. **(C)** The survival status and time in high-risk and low-risk groups. **(D)** The expression profiles of the five genes in high-risk and low-risk groups. **(E)** Time-dependent ROC curves for the five-gene model to predict patient survival. **(F)** Kaplan-Meier analysis of the five-gene risk score model to predict patient survival. Upper: Kaplan-Meier curve of the overall survival between the high-risk and low-risk groups. Lower: the number of patients at risk in the high-risk and low-risk groups at different time points. **(G)** Kaplan-Meier survival plots of the five prognostic genes *CSGALNACT1*, *REPIN1*, *TAPBPL*, *DDX11*, and *FAM53B* for multiple myeloma patients.

To further screen hub genes related to the prognosis of MM, we performed univariate and multivariate Cox regression analysis for these 13 genes. Multivariate Cox regression for the 13-gene model showed that *CSGALNACT1*, *REPIN1*, *TAPBPL*, *DDX11*, and *FAM53B* were significantly associated with the overall survival of MM patients (Figure 3F), as well as the results in univariate Cox regression analysis in Supplementary Table 1. *CSGALNACT1*, *TAPBPL*, and *FAM53B* (hazard ratio < 1) were protective genes and *REPIN1*, *DDX11* were harmful genes (hazard ratio > 1). In order to optimize the survival model, we tried to use these five genes to build a new five-gene risk score model. Multivariate Cox regression analysis for the five-gene model showed that all five genes were significantly associated with the prognosis of MM (Figure 4A). MM patients were divided into the high-risk group and the low-risk group according to the cut-off value of the five-gene risk score (Figure 4B). Similar to the 13-gene model, the number of deaths in the high-risk group was larger than that in the low-risk group (Figure 4C) and the heatmap showed the expression tendency of *CSGALNACT1*, *TAPBPL*, *FAM53B*, *REPIN1*, and *DDX11* (Figure 4D). K-M curves and time-dependent ROC curves based on the five-gene risk score model also showed favorable performance (Figures 4E,F). K-M analyses for the individual predictive power of these five genes showed that high expressions of *REPIN1* and *DDX11* were infaust for the survival outcome of MM, but high expressions of *CSGALNACT1*, *TAPBPL*, and *FAM53B* were beneficial for the survival outcome (Figure 4G).

GSE9782 dataset was used to validate the established five-gene risk score model. Multivariate Cox regression analysis showed that *REPIN1* and *FAM53B* were significantly correlated with the overall survival of MM patients (Supplementary Figure 2A). High-risk group and low-risk group of MM patients were also obtained (Supplementary Figures 2B,C). The expression of *REPIN1* was upregulated in the high-risk group, while the expression of *FAM53B* and *DDX11* was downregulated. The expressions of *TAPBPL* and *CSGALNACT1* seemed to have no significant difference between the high-risk group and low-risk group (Supplementary Figure 2D). Time-dependent ROC analysis showed that the AUC value of 1-year survival was 0.667, and the AUC value of 2-year survival was 0.671 (Supplementary Figure 2E). Consistent with the training dataset GSE24080, high-risk group also predicted poor survival (log-rank *p* < 0.0001) in GSE9782 dataset (Supplementary Figure 2F). In addition, K-M survival plots of the five prognostic genes indicated that *REPIN1* (log-rank *p* = 0.0024) and *FAM53B* (log-rank *p* = 0.00012) had favorable discriminatory ability (Supplementary Figure 2G).

### Identification of Clinical Risk Indicators

**FIGURE 5:**
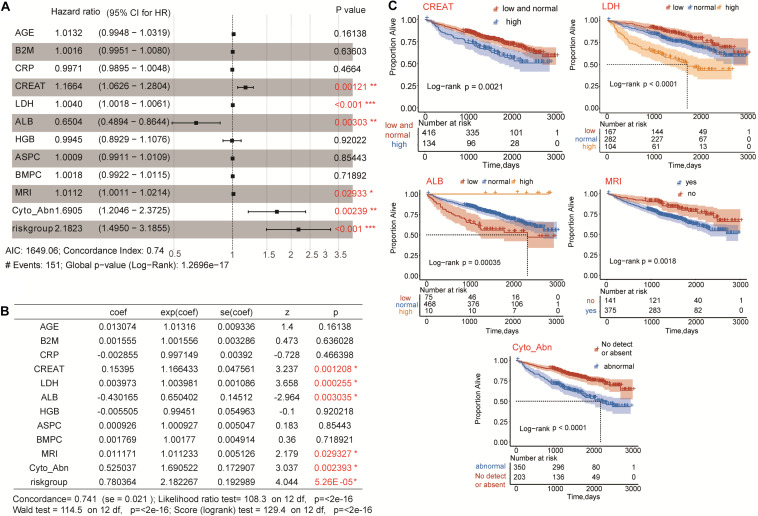
Multivariate Cox regression analysis of five-gene risk group and clinical risk indicators. **(A,B)** CREAT, LDH, ALB, MRI, Cyto_Abn, and five-gene risk group were significantly associated with the overall survival of multiple myeloma patients (*p < 0.05, **p < 0.01, and ***p < 0.001). **(C)** Kaplan-Meier survival plots of CREAT, LDH, ALB, MRI, and Cyto_Abn in multiple myeloma patients. Log-rank *p* < 0.05 was considered statistically significant.

Univariate Cox regression was conducted to analyze all the clinical indicators and the five-gene risk group (Table 1). Eleven clinical indicators and the five-gene risk group were significantly associated with the overall survival of MM patients. Further multivariate Cox regression analysis showed that four clinical risk indicators including CREAT, LDH, MRI, Cyto_Abn, and the five-gene risk group were statistics significantly risk indicators for OS prediction, while ALB was protective indicators (Figures 5A,B and Table 2). K-M survival plots showed that high levels of CREAT, LDH and low level of ALB predicted shorter overall survival of MM patients. MRI and cytogenetic abnormalities predicted shorter overall survival as well (Figure 5C).

**TABLE 1:** Univariate Cox regression analysis of all clinical indicators and the five-gene risk group.

**Clinical indicator**	**Beta**	**HR**	**95% CI for HR**	**Wald.test**	***P* value**
PROT	−0.21	0.81	0.57–1.2	1.2	0.27
AGE	0.025	1	1–1	8.8	0.0029*
SEX	−0.05	0.95	0.7–1.3	0.1	0.75
RACE	0.041	1	0.65–1.7	0.03	0.86
ISOTYPE	−0.01	0.99	0.88–1.1	0.03	0.87
B2M	0.73	2.1	1.4–3.1	12	0.00051*
CRP	0.33	1.4	1–1.9	4.1	0.044*
CREAT	0.5	1.6	1.2–2.3	9.4	0.0021*
LDH	0.0063	1	1–1	51	9.60E-13*
ALB	−0.71	0.49	0.35–0.7	16	7.00E-05*
HGB	−0.38	0.68	0.49–0.95	5.3	0.022*
ASPC	0.01	1	1–1	10	0.0015*
BMPC	0.0094	1	1–1	10	0.0016*
MRI	0.017	1	1–1	14	0.00016*
CPS1	−0.05	0.95	0.7–1.3	0.1	0.75
CPR1	−0.23	0.79	0.58–1.1	2.2	0.13
Cyto_Abn	0.82	2.3	1.7–3.1	28	1.10E-07*
Riskgroup	1.2	3.2	2.3–4.6	45	2.10E-11*

HR, hazard ratio; CI, confidence interval; and *p < 0.05.

**TABLE 2:** Multivariate Cox regression analysis of eleven clinical indicators and the five-gene risk group.

**Clinical indicator**	**Beta**	**HR**	**95% CI for HR**	***P* value**
AGE	0.013074	1.01316	0.9948–1.0319	0.16138
B2M	0.001555	1.001556	0.9951–1.0080	0.636028
CRP	–0.002855	0.997149	0.9895–1.0048	0.466398
CREAT	0.15395	1.166433	1.0626–1.2804	0.001208*
LDH	0.003973	1.003981	1.0018–1.0061	0.000255*
ALB	–0.430165	0.650402	0.4894–0.8644	0.003035*
HGB	–0.005505	0.99451	0.8929–1.1076	0.920218
ASPC	0.000926	1.000927	0.9911–1.0109	0.85443
BMPC	0.001769	1.00177	0.9922–1.0115	0.718921
MRI	0.011171	1.011233	1.0011–1.0214	0.029327*
Cyto_Abn	0.525037	1.690522	1.2046–2.3725	0.002393*
Riskgroup	0.780364	2.182267	1.4950–3.1855	5.26E-05*

HR, hazard ratio; CI, confidence interval; *p < 0.05.

*Concordance-index = 0.741 (se = 0.021).*

*Likelihood ratio test = 108.3, *p* = < 2e-16.*

*Wald test = 114.5, *p* = < 2e-16.*

*Score (logrank) test = 129.4, *p* = < 2e-16.*

### Interactive Nomogram Based on the Five-Gene Risk Score Model and Clinical Risk Indicators

**FIGURE 6:**
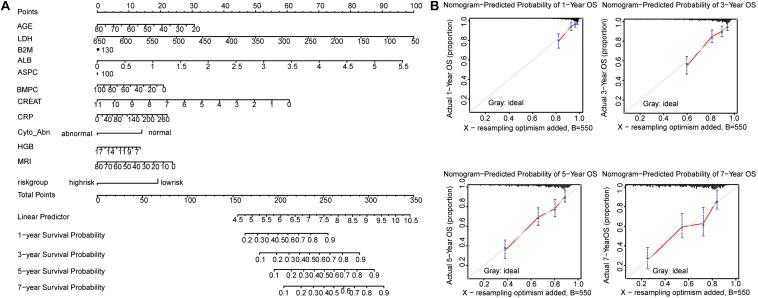
Nomogram predicting 1-year, 3-year, 5-year, and 7-year overall survival of multiple myeloma patients. **(A)** The nomogram consists of the eleven clinical risk indicators and five-gene risk group. Add the points from these twelve variables together to find the location of the Total Points. The Total Points projected on the bottom scales indicate the probability of 1-year, 3-year, 5-year, and 7-year overall survival. **(B)** The calibration curves for predicting 1-year, 3-year, 5-year, and 7-year overall survival. The *Y*-axis represents actual overall survival, the *X*-axis represents the nomogram-predicted overall survival. The gray line indicated that prediction agrees with actuality. Error bars represent 95% confidence intervals.

We developed a nomogram to predict the survival probability of MM patients using the GSE24080 dataset. The eleven clinical risk indicators (AGE, B2M, CRP, CREAT, LDH, ALB, HGB, ASPC, BMPC, MEI, and Cyto_Abn) and the five-gene risk group as variables were included in the nomogram (Figure 6A). And the C-index of this model for evaluation of OS was 0.741. The calibration plots for 1 to 7-year survival predictions were nearly coincident with the gray line, indicating that this nomogram performed well to predict the overall survival of MM patients (Figure 6B). The equation of total points was calculated by the R packages “nomogramEx” and showed in the Supplementary Table 2. To further confirm that the addition of SE-associated genes improves predictive potential, we also constructed a nomogram with only clinical data, and calibration curves were also plotted. As a result, the C-index was 0.714, which was less than 0.741, and the calibration curves were not ideal either (Supplementary Figures 3A,B).

**FIGURE 7:**
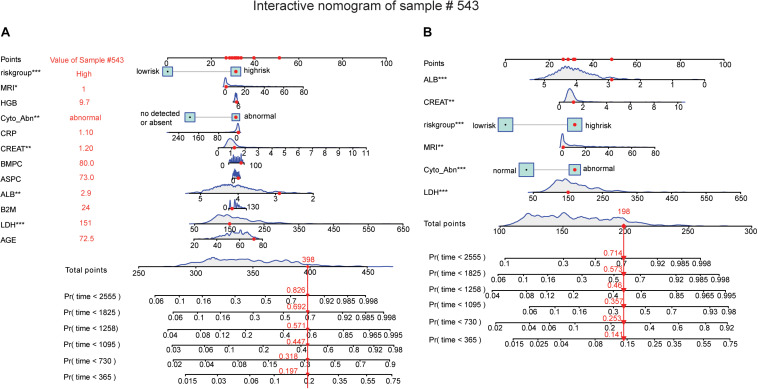
Nomogram predicting the probability of 1-year, 3-year, 5-year, and 7-year overall survival for sample GSM592937. **(A)** Eleven clinical risk indicators and five-gene risk group predicting the 1 to 7-year overall survival probability. **(B)** Five clinical risk indicators and five-gene risk group predicting the 1 to 7-year overall survival probability (*p < 0.05, **p < 0.01, and ***p < 0.001).

To display the interactive effect of nomogram, we randomly selected the sample GSM592937 of the GSE24080 dataset to validate the established nomogram. All the points were added together from the eleven clinical indicators and the five-gene risk group, therefore, the total points were 398. The probability of 1-year, 3-year, 5-year, and 7-year overall survival for sample GSM592937 were 0.197, 0.447, 0.692, and 0.826, respectively. Actually, the patient died at 1258 days, and the predictive probability of death at this time point was 0.571 (Figure 7A). Similarly, when we used the five clinical indicators and the five-gene risk group, the total points was 198. On this occasion, the probability of 1-year, 3-year, 5-year, and 7-year overall survival for sample GSM592937 were 0.141, 0.357, 0.573, and 0.714, respectively. The predictive probability of death at the time point 1258 days was 0.46, which was lower than the twelve variables (Figure 7B). Therefore, compared to six variables, twelve variables had more accurate predictive performance of the overall survival. In addition, total points with only eleven clinical indicators were also calculated and the results presented the relatively weaker prediction power (the predictive probability of death at the time point 1258 days was 0.514) compared to twelve variables containing not only clinical data, but also the five-gene risk group (Supplementary Figure 4).

## Discussion

Multiple myeloma is the world’s second most common hematologic malignancy with high heterogeneity (Ronca et al., 2020). Currently, various prognostic models have been established to guide risk stratification and predict patient survival (Kyrtsonis et al., 2009), but few of them were incorporated into clinical practice. Besides, due to those different patients were enrolled in the predictive model and different clinical parameters were statistically evaluated, the prognostic genes are so different in these models for MM. Therefore, mining powerful prognostic genes via integrated omics is still needed.

Univariate and multiple Cox regressions are widely used for survival analyses. A ten-gene risk score model (*C17orf97*, *GALNT10*, *RPP30*, *KIFC1*, *HCFC2*, *BC043172*, *TSPAN13*, *DNAJB9*, *FBN1*, *and EIF4G2*) and a four-lncRNA prognostic risk model (*RP4-803 J11.2*, *RP1-43E13.2*, *RP11-553 L6.5*, *and ZFY-AS1*) were established to predict the OS of MM patients (Zhou et al., 2015; Hu et al., 2016). Recently, Lasso penalized Cox regression is popular among researchers and it could minimize overfitting. A nine-gene prognostic signature (*HLA-DPB1*, *TOP2A*, *FABP5*, *CYP1B1*, *IGHM*, *FANCI*, *LYZ*, *HMGN5*, and *BEND6*) related to the ISS stage of MM was developed via weighted gene co-expression network analysis (WGCNA) and Lasso (Liu et al., 2018). A 16-gene (*ATIC*, *BNIP3L*, *CALCOCO2*, *DNAJB1*, *DNAJB9*, *EIF4EBP1*, *EVA1A*, *FKBP1B*, *FOXO1*, *FOXO3*, *GABARAP*, *HIF1A*, *NCKAP1*, *PRKAR1A*, *TM9SF1*, and *SUPT20H*) prognostic model related to autophagy was also established by Lasso (Zhu et al., 2019). Moreover, a six-gene risk score model (*ZNF486*, *EPHA5*, *RP11.326C3.15*, *DUSP6*, *DUSP10*, and *TRIAP1*) for the prognostic prediction of PI-treated myeloma patients was developed by the random survival forest variable hunting (RSF-VH) algorithm (Liu et al., 2019). Interestingly, Chen Sun et al. developed the current state-of-the-art prognostic model for MM via a complete hazard-ranking algorithm called GuanRank with Gaussian process regression (GPR), which seemed to be more accurate than Cox model and random survival forests, despite several limitations (Sun et al., 2019). However, these previous studies have not reported a prognostic model based on SEs-associated genes, which were found to regulate the expression of hub genes associated with MM tumorigenesis (Jin et al., 2018). In addition, clinical indicators were rarely integrated with gene expression signature for the prediction of survival.

In the present study, we performed the Lasso penalized Cox regression analysis for screening the potential SE-associated genes and establishing the gene risk score model to predict the OS of MM patients. Besides, we included the gene risk group and clinical indicators in our nomogram to accurately predict patient survival. A total of 410 SE-associated genes were analyzed by Lasso penalized Cox regression in our study, and 35 and 13 genes were firstly screened using the parameter of lambda.min and lambda.lse. Considering the predictive performances of two predictive models were not obviously different according to AUC and Wilcoxon test, we further investigated the 13-gene model containing fewer genes compared to the 35-gene model. We constructed a risk score model based on the expressions of 13 genes to determine whether these hub SE-associated genes affect the OS of MM patients. The low-risk group was significantly correlated with improved survival outcome according to the result of the K-M survival curve and AUC. Through further univariate and multivariate Cox regression analysis, five statistically significant genes were screened. We found that the established five-gene risk model performed well both in the training set and the validation set. For the training set GSE24080, *CSGALNACT1*, *FAM53B*, and *TAPBPL* were significantly downregulated, while *REPIN1* and *DDX11* were significantly upregulated in high-risk MM patients. In the validation set GSE9782, *FAM53B* and *REPIN1* were expressed consistently with that in the training set. These two genes also showed improved predictive performance independently, both in the training set and validation set, indicating that *FAM53B* and *REPIN1* may act as independent prognostic factors for MM patients.

*FAM53B* is a new vertebrate gene controlling cell proliferation (Thermes et al., 2006). In acute lymphoblastic leukemia (ALL) cells, the *FAM53B* fusion transcript could code a truncated FAM53B protein, which was considered to be of great biological significance because of its entire conserved domain, even though the mechanism was unclear (Panagopoulos et al., 2017). A four-gene model of MM consisting of *FAM53B*, *KIF21B*, *WHSC1*, and *TMPO* could predict the overall survival of MM patients independently, and the expression of *FAM53B* was downregulated in high-risk patients compared to low-risk patients. Our study also suggested *FAM53B* as an independent prognostic factor, but further analysis and experiments are needed to validate.

Replication Initiator 1 (REPIN1) is a zinc finger protein involved in DNA binding and bending (Klöting et al., 2007; Heiker and Klöting, 2013). It was believed that *REPIN1* could contribute to breast cancer tumorigenesis (Rengasamy et al., 2017). However, high *REPIN1* expression was found to inhibit cell proliferation, migration and invasion in glioma cells (Wang and Lin, 2018). In our study, we found that high expression of *REPIN1* may act as a prognostic factor for the inferior outcome of MM, but further functional studies are needed.

*Chondroitin sulfate N-acetylgalactosaminyltransferase 1* (*CSGALNACT1*) was one of the most frequently lost genes in oral squamous cell carcinoma (OSCC; Yong et al., 2014) and follicular variant of papillary thyroid carcinoma (FVPTC; Schulten et al., 2015). On the contrary, *CSGALNACT1* could act as a risk factor in breast cancer (Iida et al., 2015) and prostate cancer (Munkley et al., 2016; Munkley, 2017). As for MM, Bret et al. (2009) found that the expression of *CSGALNACT1* in MM cells was lower than that in normal bone marrow plasma cells, thus, high expressions of *CSGALNACT1* may correlate with a good prognosis in MM patients. More importantly, Agnelli et al. (2011) found that the neighbor-gene model, comprising of *CSGALNACT1* and *SLC7A7* could predict overall survival independently in MM patients. Compared to low-risk patients, high-risk patients had lower level of *CSGALNACT1* expression, which was consistent with our studies.

Major histocompatibility complex class I (MHC I) plays an important role in immunosurveillance by presenting antigenic peptides to T cells (Ilca et al., 2018a). TAP binding protein-like (TAPBPL) was a novel peptide editor for MHC I (Neerincx and Boyle, 2017). Interestingly, Ilca et al. (2018b) found that soluble TAPBPL could efficiently load antigenic peptides onto breast cell line MCF-7 for recognition by CD8 + T cells, indicating that *TAPBPL* gene may contribute to the targeted therapy of tumors by loading antigenic peptides onto tumor cells including MM cells.

*DEAD/H-box helicase 11* (*DDX11*) plays an important role in cellular replication and DNA repair (Bharti et al., 2014). Recently, *DDX11* was considered to have carcinogenic potential in clear cell renal cell carcinoma (ccRCC), lung adenocarcinoma (ADC), and melanoma (Bhattacharya et al., 2012; Li et al., 2019; Park et al., 2020a,b), despite that its specific roles and mechanisms in cancers are unclear (Petrakis et al., 2016). In the present study, the expression of *DDX11* in the validation set of GSE24080 was opposite to the expression in the training set of GSE9082. One plausible reason for this discrepancy is that the baseline and clinical characteristics of MM patients included in the GSE24080 dataset and GSE9782 dataset were different to some extent.

The inclusion of clinical indicators usually improves the performance of prognostic models. In the present study, we also performed a nomogram consisting of the five-gene risk group and eleven clinical indicators, which may help to predict the 1-year, 3-year, 5-year, and 7-year overall survival of MM patients. As a result, high level of CREAT and LDH may predict poor overall survival, while high level of ALB predicts good overall survival. MRI and cytogenetic abnormalities may predict shorter survival as well. Besides, our study revealed that the more clinical indicators included, the higher the accuracy of the nomogram may be. More importantly, we confirmed that the addition of SE-associated five-gene risk group improved predictive power (C-index was 0.741) compared to the nomogram model with only clinical data (C-index was 0.714). Therefore, our established nomogram may be useful to predict the overall survival of MM patients clinically. Since *CSGALNACT1*, *FAM53B*, *TAPBPL*, *REPIN1*, and *DDX11* are SE-associated genes, and their roles have not been elucidated in MM, we recommend conducting functional studies of these five genes in the near future, and the in-depth evaluation of the five-gene risk model in prospective cohorts are also needed.

## Conclusion

We established a five-gene risk score model to predict the overall survival of MM patients by Lasso regression, univariate and multivariate Cox regression analyses. The nomogram comprising the five-gene risk group and eleven clinical indicators may be helpful for prognostic prediction of MM patients. In addition, *CSGALNACT*, *FAM53B*, *TAPBPL*, *REPIN1*, and *DDX11* are potential SE-associated genes for MM, which may play a vital role in the development and progression of MM.

## Data Availability Statement

The datasets presented in this study can be found in online repositories. The names of the repository/repositories and accession number(s) can be found in the article/Supplementary Material.

## Author Contributions

QQ contributed to the conception and design of the study. TQ and JQ analyzed and wrote the first draft of the manuscript. CT, QL, GL, and JW revised the manuscript. All authors contributed to manuscript revision, read, and approved the submitted version.

## Conflict of Interest

The authors declare that the research was conducted in the absence of any commercial or financial relationships that could be construed as a potential conflict of interest.

## Footnotes

1 https://www.ncbi.nlm.nih.gov/geo/

2 http://metascape.org/gp/index.html#/main/step1

3 http://dbcorc.cam-su.org

## Abbreviations

ALB: Albumin
ALL: Acute lymphoblastic leukemia
ASPC: Aspirate plasma cells
AUC: Area under the ROC
B2M: Beta-2 microglobulin
BMPC: Bone marrow biopsy plasma cells
ccRCC: Clear cell renal cell carcinoma
C-index: Concordance-index
CPR1: Clinical parameter R1
CPS1: Clinical parameter S1
CREAT: Creatinine
CRP: C-reactive protein
CSGALNACT1: Chondroitin sulfate N-acetylgalactosaminyltransferase 1
Cyto_Abn: Cytogenetic abnormalities
DDX11: DEAD/H-box helicase 11
ECG: Electrocardiographic
FVPTC: Follicular variant of papillary thyroid carcinoma
GEO: Gene Expression Omnibus
GPR: Gaussian process regression
HGB: Haemoglobin
IMiD: Immunomodulatory drugs
ISS: International Staging System
K-M: Kaplan-Meier
Lasso: Least absolute shrinkage and selection operator
LDH: Lactate dehydrogenase
LUAD: Lung adenocarcinoma
MHC I: Major histocompatibility complex class I
MM: Multiple myeloma
MRI: Magnetic resonance imaging
NLR: Neutrophils to lymphocytes ratio
OS: Overall survival
OSCC: Oral squamous cell carcinoma
PFS: Progression-free survival
PI: Proteasome inhibitors
PLR: Platelets to lymphocytes ratio
PROT: Protocol
REPIN1: Replication Initiator 1
R-ISS: Revised International Staging System
ROC: Receiver operating characteristic
RSF-VH: Random survival forest variable hunting
SE: Super-enhancer
TAPBPL: TAP binding protein-like
TF: Transcription factor
WGCNA: Weighted gene co-expression network analysis.
