# Supplementary material for: Super-Enhancer Associated Five-Gene Risk Score Model Predicts Overall Survival in Multiple Myeloma Patients
Source: Front Cell Dev Biol. 2020 Dec 3;8:596777. doi: 10.3389/fcell.2020.596777 (PMC7744621; doi:10.3389/fcell.2020.596777)
Supplement: Supplementary file 1 [file Table_1.DOCX]

Supplementary Material

# Supplementary Figures and Tables

## Supplementary Figures


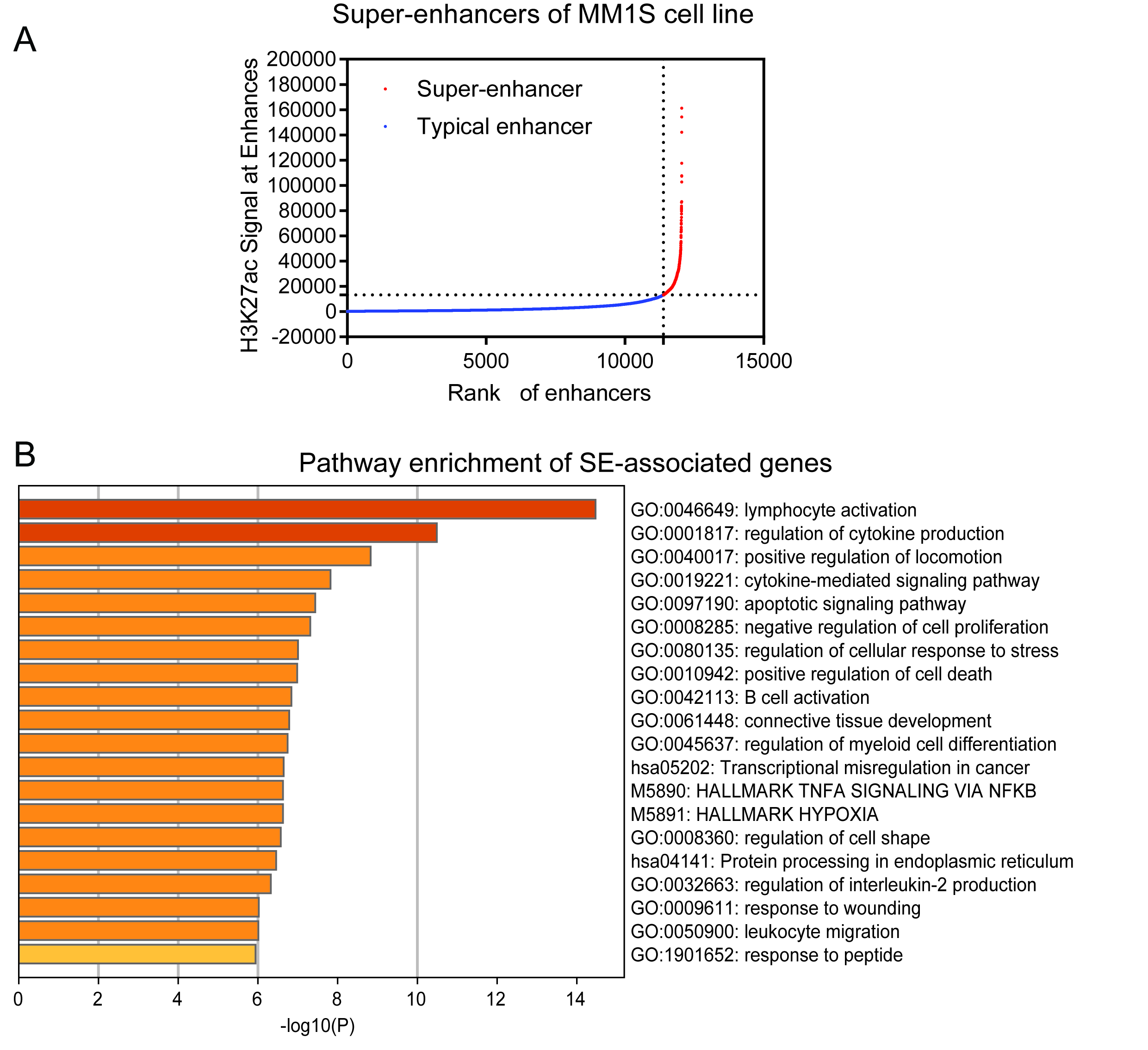


**Supplementary Figure 1.** Pathway enrichment analysis of SE-associated genes. **(A)** Identification of super-enhancers in MM1S cell line by ROSE algorithm. Enhancers were ranked and plotted by H3K27ac ChIP-seq signal. Red dots represent super-enhancers. Blue dots represent typical enhancers. **(B)** Pathway enrichment analysis of SE-associated genes in MM1S cell line was performed on the website (<http://metascape.org/gp/index.html#/main/step1>).


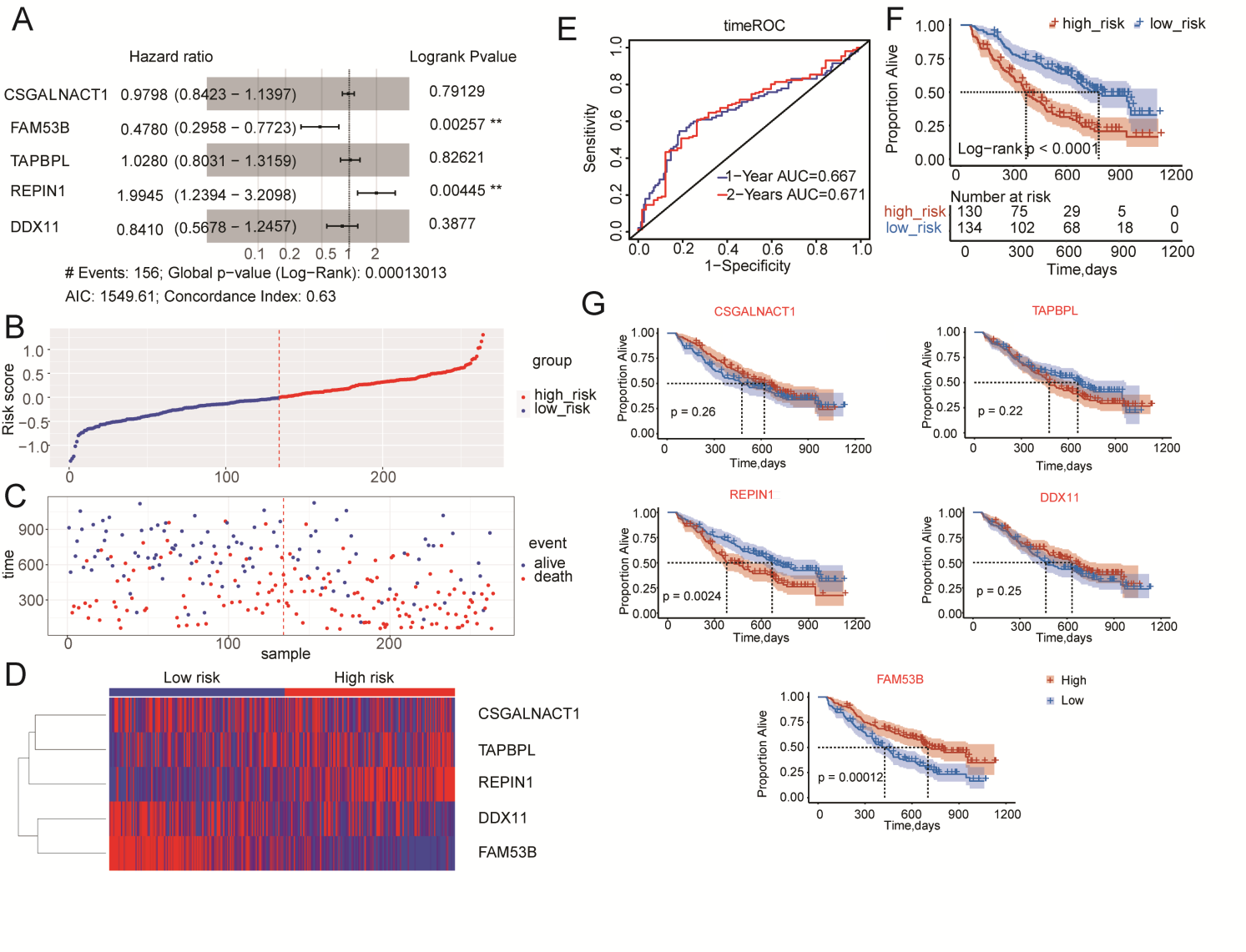


**Supplementary Figure 2.** Validation of the five-gene risk score model in the GSE9782 dataset. **(A)** Multivariate Cox regression analysis of the five genes (***p*<0.01). **(B)** Five-gene risk score distribution. Multiple myeloma patients were divided into the high-risk group and low-risk group based on the cut-off value. **(C)** The survival status and time in high-risk and low-risk groups. **(D)** The expression profiles of the five genes in high-risk and low-risk groups. **(E)** Time-dependent ROC curves for the five-gene model to predict patient survival. **(F)** Kaplan-Meier analysis of the five-gene risk score model to predict patient survival. Upper: Kaplan-Meier curve of the overall survival between the high-risk and low-risk groups. Lower: the number of patients at risk in the high-risk and low-risk groups at different time points. **(G)** Kaplan‑Meier survival plots of the five prognostic genes CSGALNACT1, REPIN1, TAPBPL, DDX11 and FAM53B for multiple myeloma patients. High expression of REPIN1 and low expression of FAM53B were correlated with poor survival.


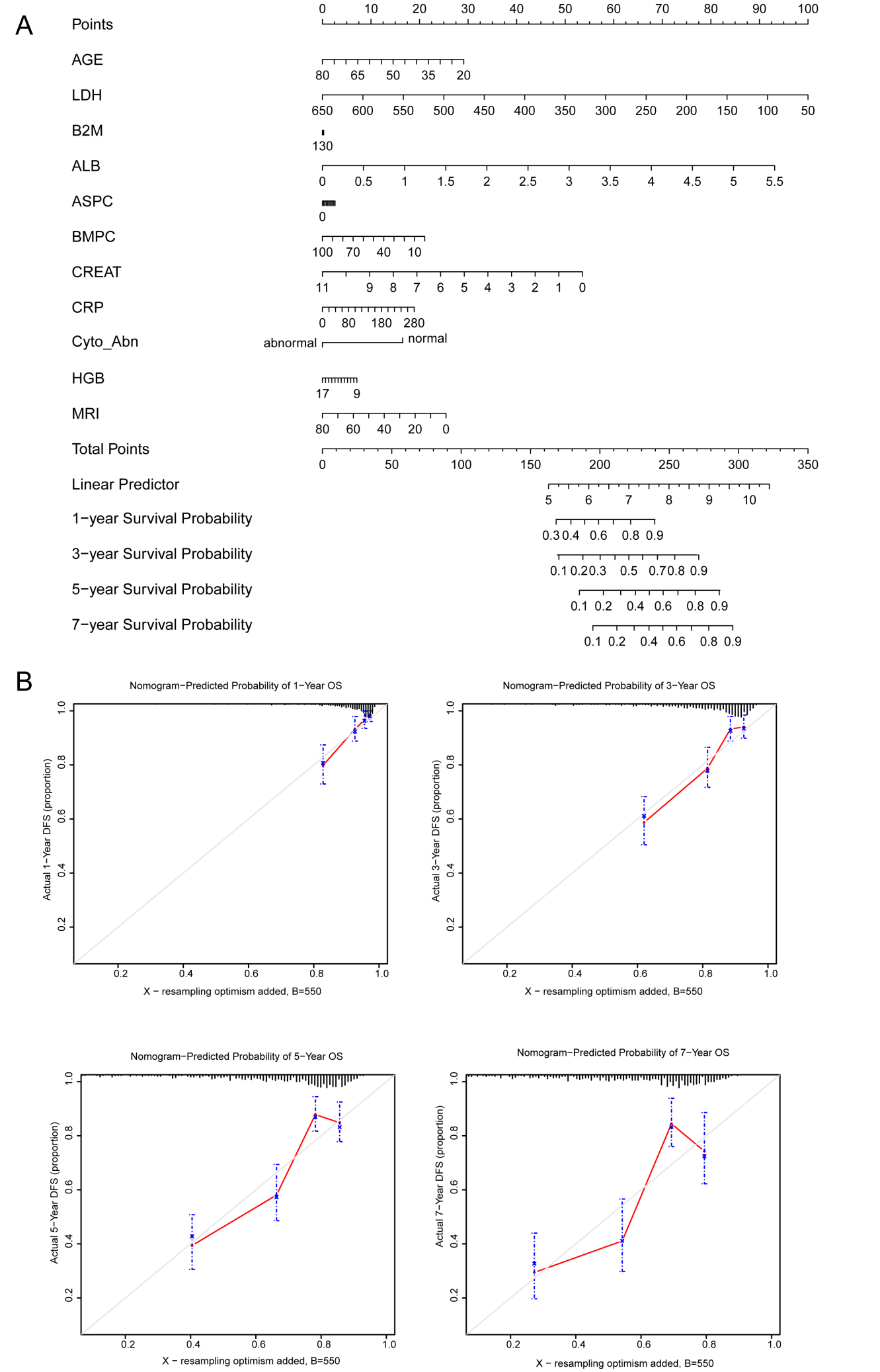


**Supplementary Figure 3.** Nomogram predicting the probability of 1-year, 3-year, 5-year and 7-year overall survival using only clinical data. **(A)** Eleven clinical indicators were included in the nomogram for predicting the 1-year, 3-year, 5-year and 7-year overall survival probability by adding up all the eleven points. **(B)** The Calibration curves were plotted to verify nomogram-predictive accuracy.


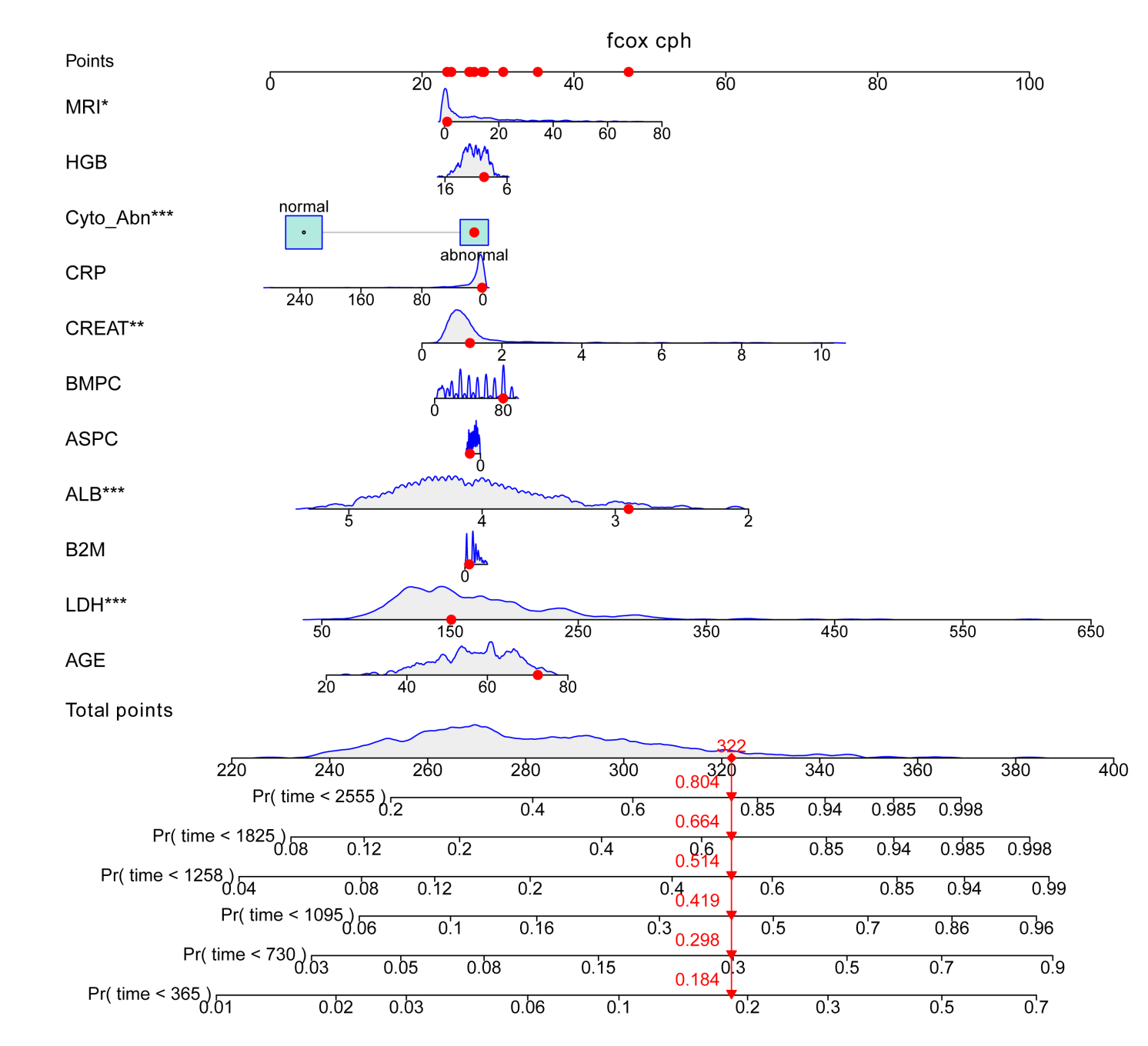


**Supplementary Figure 4.** Nomogram predicting the probability of 1 to 7-year overall survival for sample GSM592937 using only eleven clinical indicators.

## Supplementary Tables

**Supplementary Table 1.** Univariate Cox regression analysis of the 35 genes screened.

| Gene | beta | HR (95% CI for HR) | wald.test | p.value |
| --- | --- | --- | --- | --- |
| SLC9A3R1 | 4 | 54 (2.2-1300) | 6 | 0.014 |
| RASGRP3 | -1.6 | 0.21 (0.089-0.49) | 13 | 0.00035 |
| MGAT3 | -2 | 0.13 (0.035-0.52) | 8.5 | 0.0035 |
| PLA2G4A | 2.2 | 8.9 (2.4-33) | 11 | 0.00097 |
| IFI16 | 6.8 | 880 (44-17000) | 20 | 9.00E-06 |
| PDE4B | -3.4 | 0.035 (0.0083-0.14) | 21 | 3.60E-06 |
| RARA | -0.71 | 0.49 (0.23-1) | 3.5 | 0.06 |
| RAB3B | -1.1 | 0.33 (0.16-0.7) | 8.5 | 0.0036 |
| RHOQ | -3.5 | 0.031 (0.0022-0.44) | 6.6 | 0.01 |
| PTK2B | -3 | 0.051 (0.0091-0.29) | 11 | 7.00E-04 |
| PAK1 | -1.6 | 0.2 (0.055-0.75) | 5.7 | 0.017 |
| TPST2 | 2.1 | 8.1 (1.7-39) | 6.9 | 0.0086 |
| CSGALNACT1 | -2.7 | 0.065 (0.02-0.21) | 21 | 5.80E-06 |
| NLRP4 | -0.94 | 0.39 (0.21-0.74) | 8.3 | 0.0039 |
| REPIN1 | 6.1 | 460 (13-16000) | 11 | 0.00072 |
| TAPBPL | -5.3 | 0.005 (0.00058-0.043) | 23 | 1.50E-06 |
| KCNA2 | -2.7 | 0.068 (0.018-0.26) | 15 | 8.60E-05 |
| MLF1 | 1.8 | 6.1 (1.5-24) | 6.6 | 0.01 |
| GNG7 | -5.4 | 0.0045 (0.00025-0.08) | 13 | 0.00024 |
| E2F2 | 3.3 | 27 (6.9-110) | 22 | 2.50E-06 |
| PLCG2 | -5.9 | 0.0028 (0.00025-0.032) | 22 | 2.30E-06 |
| DDX11 | 5.1 | 160 (7.4-3300) | 11 | 0.0012 |
| MACC1 | 3.4 | 31 (3.4-270) | 9.4 | 0.0022 |
| FAM53B | -6.8 | 0.0011 (7.8e-05-0.017) | 25 | 6.80E-07 |
| EDEM3 | 3.6 | 35 (4-310) | 10 | 0.0014 |
| XIRP1 | 1.3 | 3.8 (1.4-11) | 6.4 | 0.011 |
| ARID5B | -2.8 | 0.062 (0.019-0.2) | 22 | 3.10E-06 |
| SCNN1B | -0.9 | 0.41 (0.2-0.82) | 6.4 | 0.011 |
| RAC2 | 4.1 | 60 (2.1-1700) | 5.8 | 0.016 |
| ZMYND8 | 2.1 | 8.3 (0.42-160) | 1.9 | 0.16 |
| DENND1B | 3.2 | 25 (2.9-220) | 8.5 | 0.0035 |
| STK17B | 1.3 | 3.5 (1-12) | 4.1 | 0.044 |
| LRRC2 | -0.53 | 0.59 (0.42-0.82) | 9.5 | 0.0021 |
| SMC4 | 7.6 | 2100 (110-37000) | 27 | 2.50E-07 |
| B4GALT3 | 6.1 | 440 (27-7000) | 18 | 1.70E-05 |

HR, hazard ratio; CI, confidence interval

**Supplementary Table 2.** The equation of total points using nomogram

| **Items** |  | **Input data** |  | **Points** |
| --- | --- | --- | --- | --- |
| AGE | 1 year - 80 years | **72.5** | **years** | **28.79** |
| B2M | Beta-2 microglobulin | **24** | **mg/l** | **1.565419176** |
| CREAT | Creatinine | **1.2** | **mg/dl** | **7.749719772** |
| CRP | C-reactive protein, mg/l | **1.1** | **mg/l** | **33.4048223** |
| LDH | Lactate dehydrogenase | **151** | **U/l** | **16.83333338** |
| ALB | Albumin | **2.9** | **g/dl** | **46.91584208** |
| HGB | Haemoglobin | **9.7** | **g/dl** | **1.685677958** |
| ASPC | Aspirate plasma cells | **73** | **%** | **2.835728216** |
| BMPC | Bone marrow biopsy plasma cells | **80** | **%** | **5.935284** |
| MRI | MRI-defined focal lesions (skull, spine, pelvis) | **1** | **numbers** | **0.468585567** |
| Cyto_Abn | An indicator of the detection of cytogenetic abnormalities | **1** | **1=abnormal; 0=no detected or absent** | **22.02424** |
| Riskgroup | Riskgroup based on genes | **1** | **1=high risk; 0=low risk** | **32.73466** |
|  | **Totals points** |  |  | **200.95** |
| **Results:** | 1-year Survival Prob |  |  | **0.807606144** |
|  | 3-years Survival Prob |  |  | **0.54536388** |
|  | 5-years Survival Prob |  |  | **0.313287859** |
|  | 7-years Survival Prob |  |  | **0.17983917** |

[1] "points = 0 * AGE ^3 + 0 * AGE ^2 + 0.548431256 * AGE + -10.968625111"

[2]"points = 0 * LDH ^2 + 0.166666667 * LDH + -8.333333333"

[3]"points = 0 * B2M ^2 + 0.065225799 * B2M + 0"

[4]"points = 0 * ALB ^2 + -18.044554648 * ALB + 99.245050562"

[5]"points = 0.038845592 * ASPC + 0"

[6]"points = 0 * BMPC ^2 + 0.07419105 * BMPC + 0"

[7]"points = 0 * CREAT ^2 + 6.45809981 * CREAT + 0"

[8]"points = 0 * CRP ^2 + -0.119773475 * CRP + 33.536573123"

[9] Cyto_Abn

1 22.02424

0 0.00000

[10]"points = 0 * HGB ^3 + 0 * HGB ^2 + -0.230914788 * HGB + 3.925551402"

[11]"points = 0 * MRI ^3 + 0 * MRI ^2 + 0.468585567 * MRI + 0"

[12]riskgroup

1 32.73466

0 0.00000

The total points was the sum of above points of variable.

The survival Probabilitis was calculated by the following equation:

[13]"1 year Survival Prob = 1.59e-07 * points ^3 + -0.000142436 * points ^2 + 0.033441067 * points + -1.450912656"

[14]"3 years Survival Prob = 2.75e-07 * points ^3 + -0.000185448 * points ^2 + 0.033494521 * points + -0.928332907"

[15]"5 years Survival Prob = 2.75e-07 * points ^3 + -0.000161652 * points ^2 + 0.023486122 * points + -0.110126905"

[16]"7 years Survival Prob = 2.75e-07 * points ^3 + -0.000147902 * points ^2 + 0.018328709 * points + 0.237570631"
