# Supplementary figures and images for: Super-Enhancer Associated Five-Gene Risk Score Model Predicts Overall Survival in Multiple Myeloma Patients
Source: Front Cell Dev Biol. 2020 Dec 3;8:596777. doi: 10.3389/fcell.2020.596777 (PMC7744621; doi:10.3389/fcell.2020.596777)

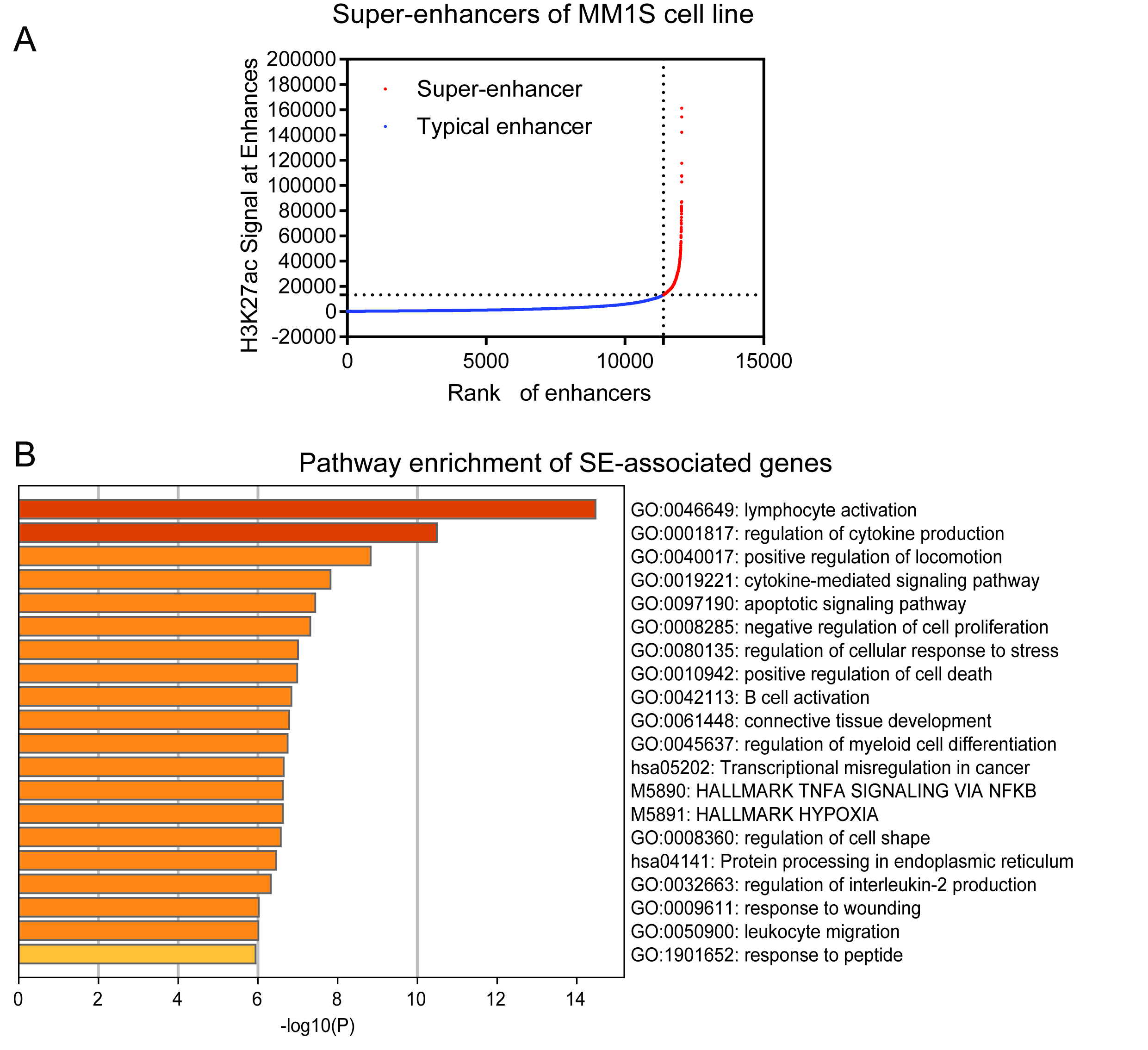

Supplement: Supplementary file 2 [file Image_1.TIF]

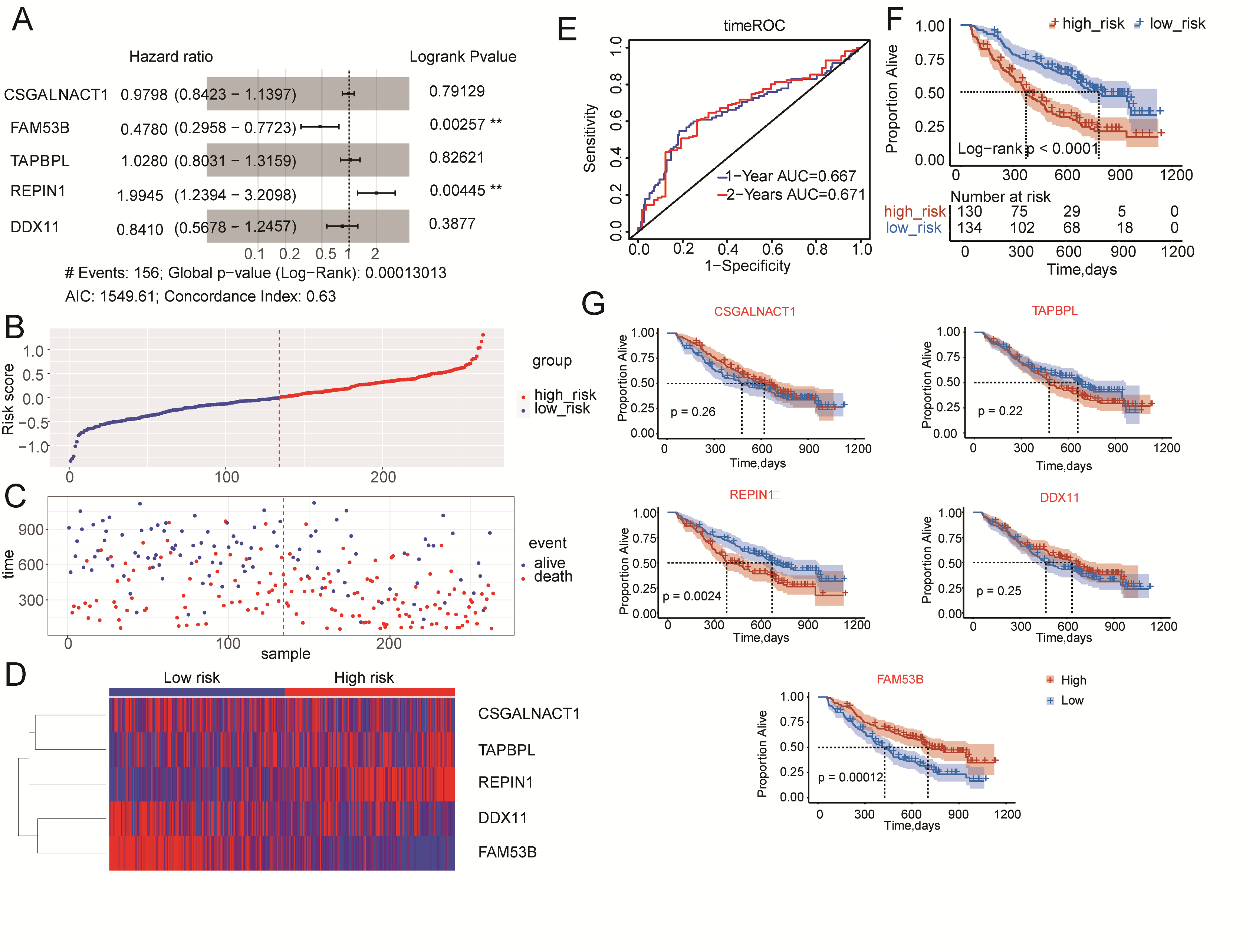

Supplement: Supplementary file 3 [file Image_2.TIF]

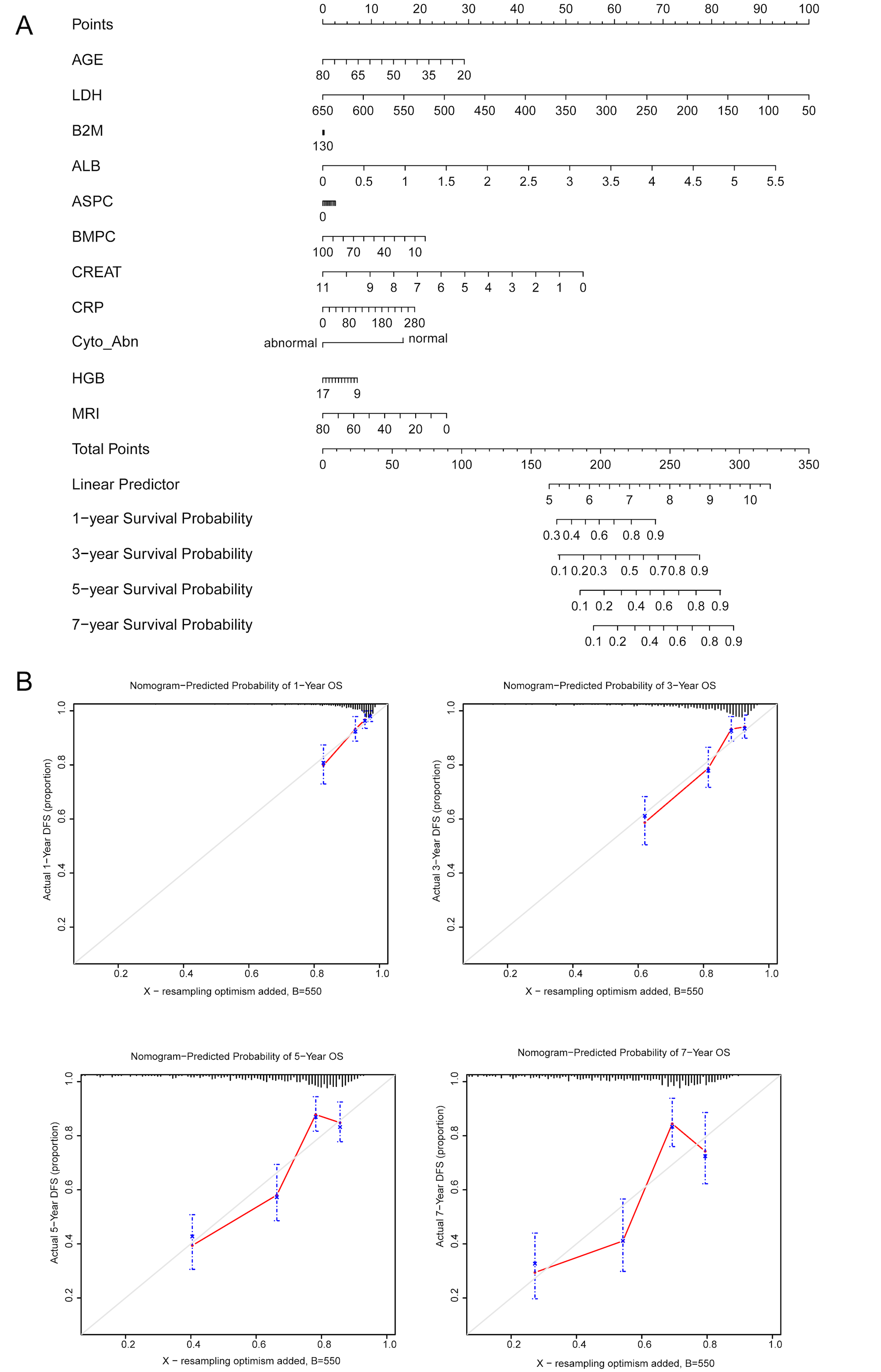

Supplement: Supplementary file 4 [file Image_3.TIF]

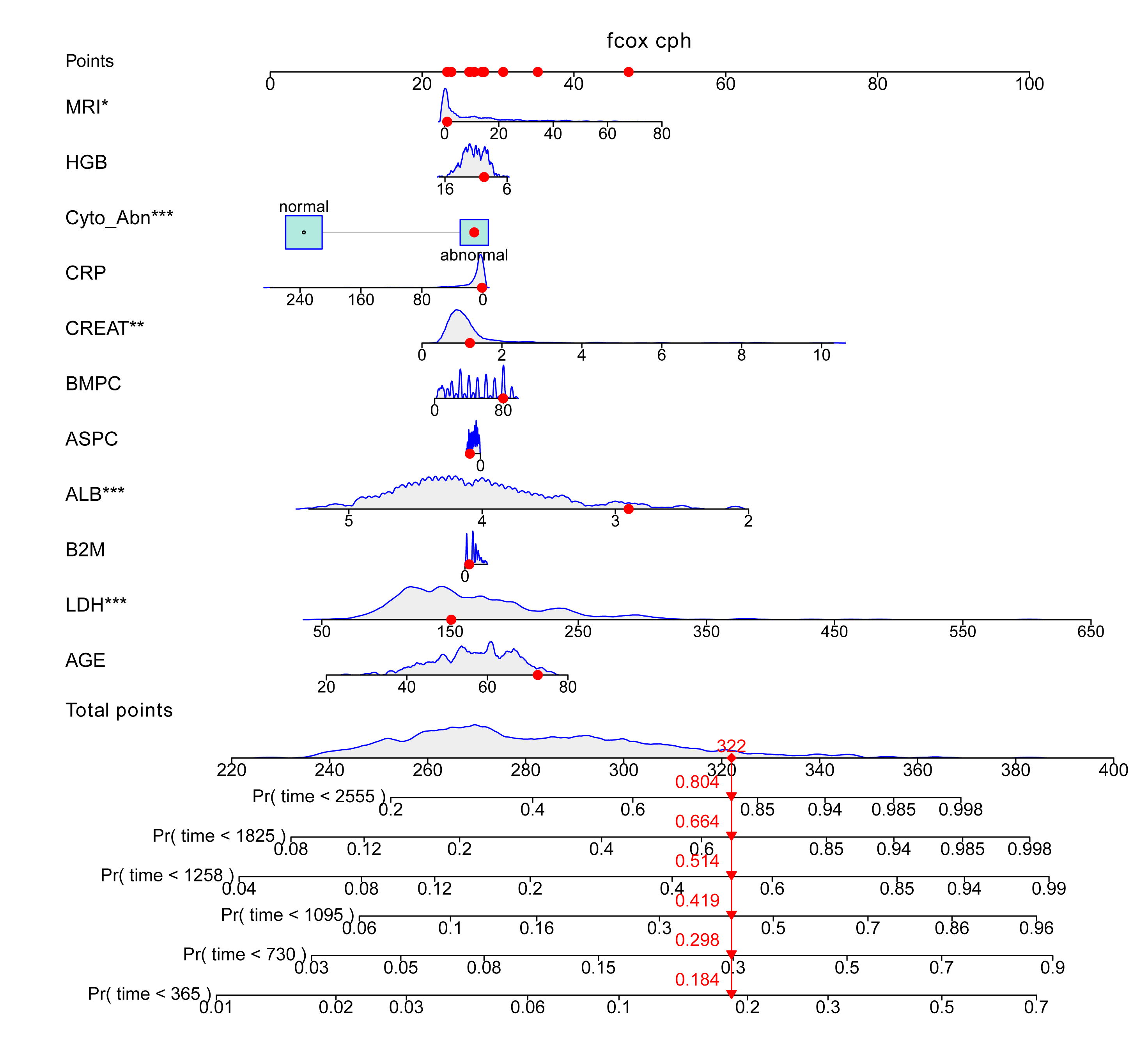

Supplement: Supplementary file 5 [file Image_4.TIF]
